# Supplementary material for: Multicompartmentalized Microvascularized Tumor-on-a-Chip to Study Tumor-Stroma Interactions and Drug Resistance in Ovarian Cancer
Source: Cell Mol Bioeng. 2024 Sep 14;17(5):345–67. doi: 10.1007/s12195-024-00817-y (PMC11538101; doi:10.1007/s12195-024-00817-y)
Supplement: Supplementary file 2 — Supplementary file2 (PPTX 13562 kb) [file 12195_2024_817_MOESM2_ESM.pptx]

## Slide 1
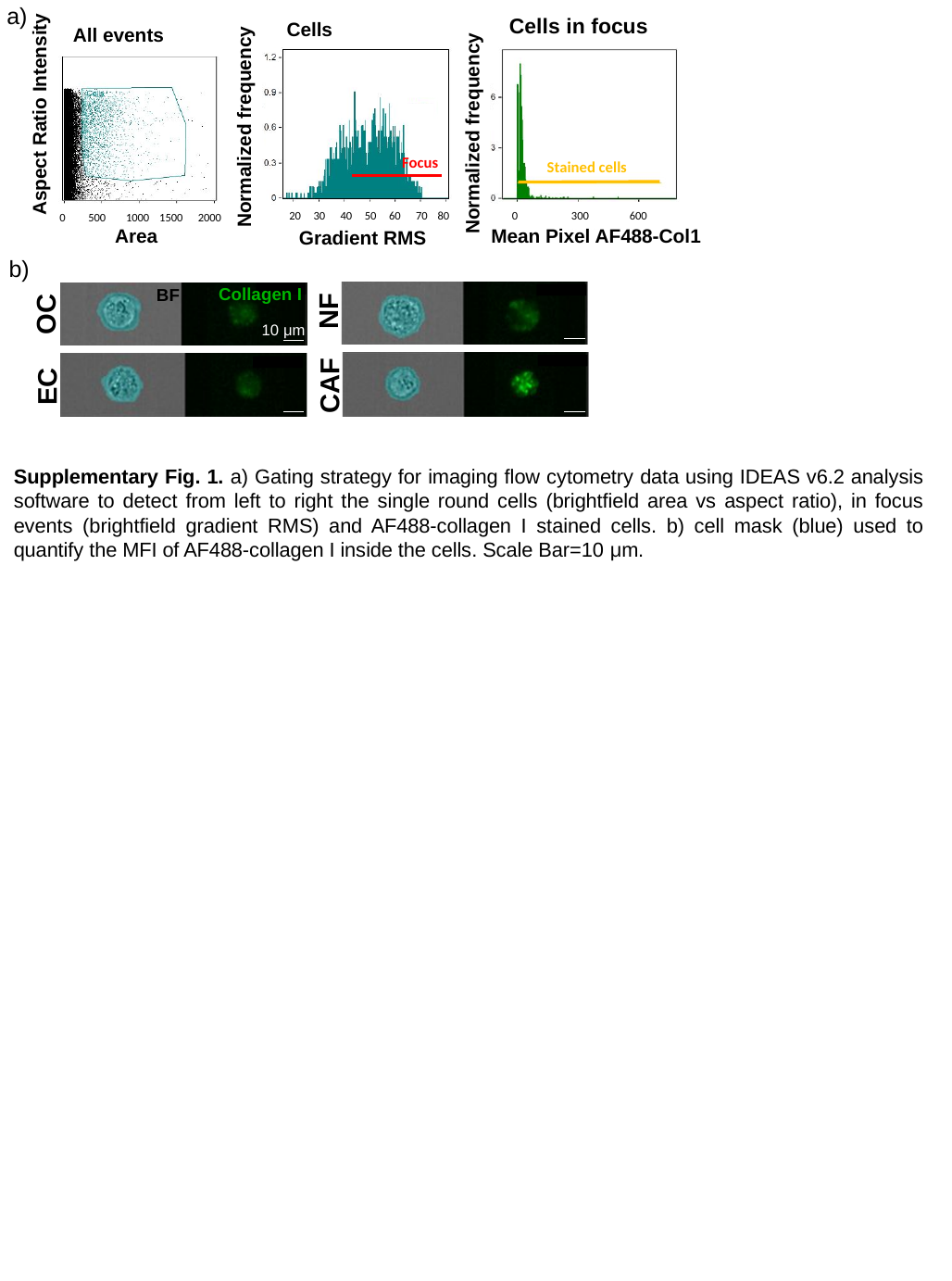

a)
Cells in focus
Cells
All events
Normalized frequency
Normalized frequency
Aspect Ratio Intensity
Focus
Stained cells
20 30 40 50 60 70 80
0 300 600
0 500 1000 1500 2000
Mean Pixel AF488-Col1
Area
Gradient RMS
b)
Collagen I
BF
NF
OC
10 μm
CAF
EC
Supplementary Fig. 1. a) Gating strategy for imaging flow cytometry data using IDEAS v6.2 analysis software to detect from left to right the single round cells (brightfield area vs aspect ratio), in focus events (brightfield gradient RMS) and AF488-collagen I stained cells. b) cell mask (blue) used to quantify the MFI of AF488-collagen I inside the cells. Scale Bar=10 μm.

## Slide 2
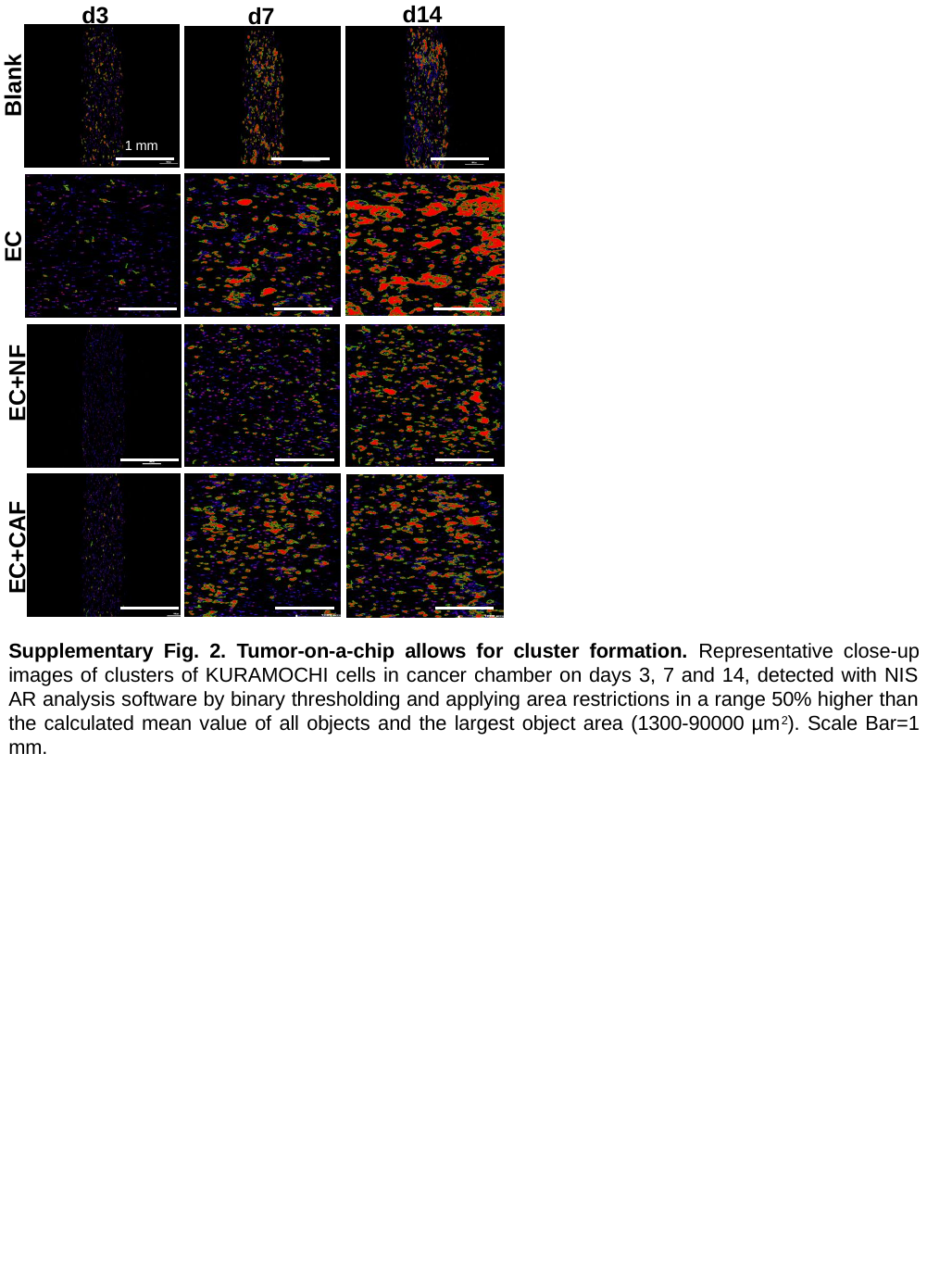

d14
d3
d7
Blank
1 mm
EC
EC+NF
EC+CAF
Supplementary Fig. 2. Tumor-on-a-chip allows for cluster formation. Representative close-up images of clusters of KURAMOCHI cells in cancer chamber on days 3, 7 and 14, detected with NIS AR analysis software by binary thresholding and applying area restrictions in a range 50% higher than the calculated mean value of all objects and the largest object area (1300-90000 µm2). Scale Bar=1 mm.

## Slide 3
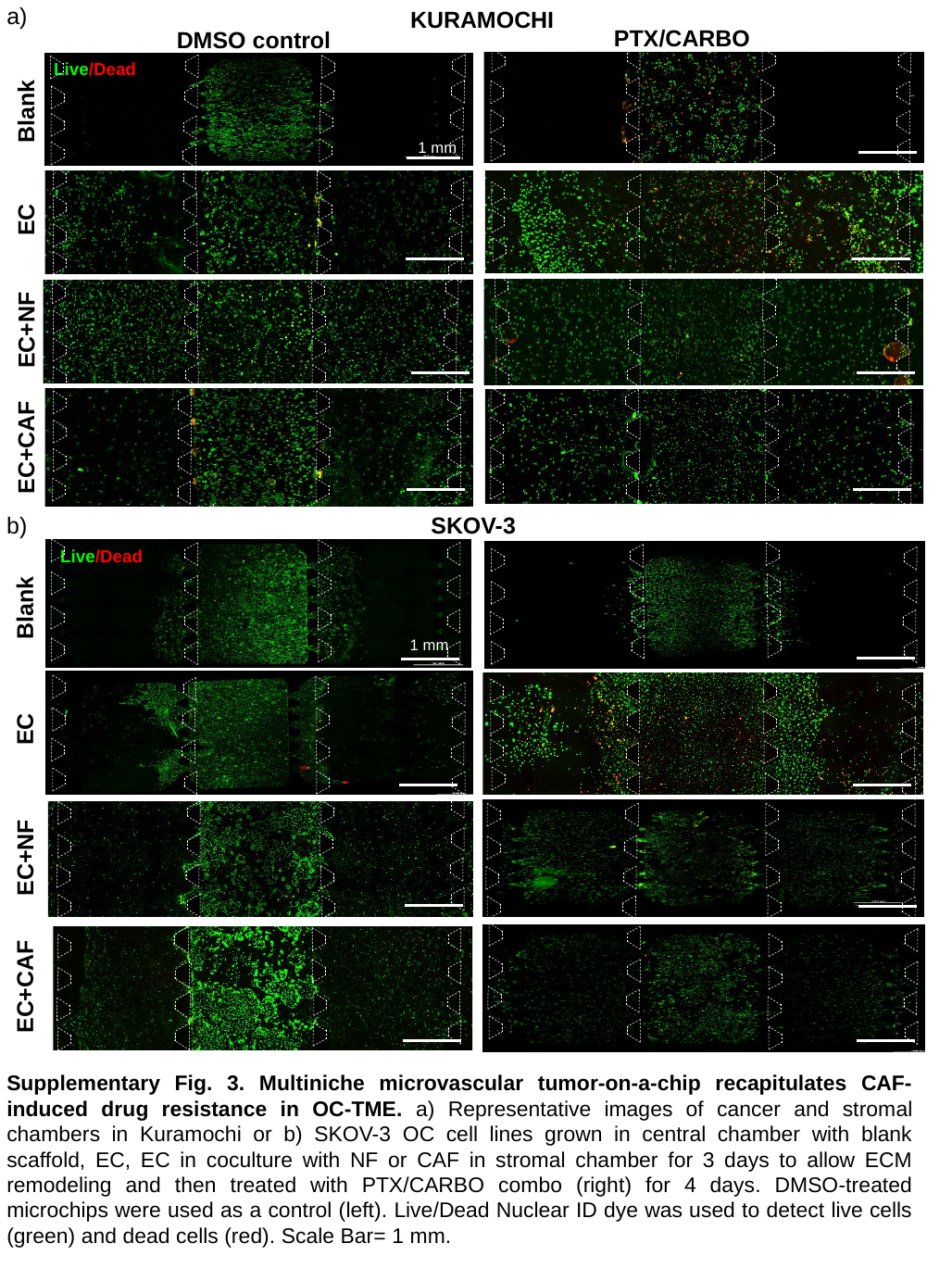

Healthy fibroblasts 090823
a)
KURAMOCHI
PTX/CARBO
DMSO control
Blank
EC
EC+NF
EC+CAF
Live/Dead
1 mm
b)
SKOV-3
Live/Dead
Blank
EC
EC+NF
EC+CAF
1 mm
Supplementary Fig. 3. Multiniche microvascular tumor-on-a-chip recapitulates CAF-induced drug resistance in OC-TME. a) Representative images of cancer and stromal chambers in Kuramochi or b) SKOV-3 OC cell lines grown in central chamber with blank scaffold, EC, EC in coculture with NF or CAF in stromal chamber for 3 days to allow ECM remodeling and then treated with PTX/CARBO combo (right) for 4 days. DMSO-treated microchips were used as a control (left). Live/Dead Nuclear ID dye was used to detect live cells (green) and dead cells (red). Scale Bar= 1 mm.

## Slide 4
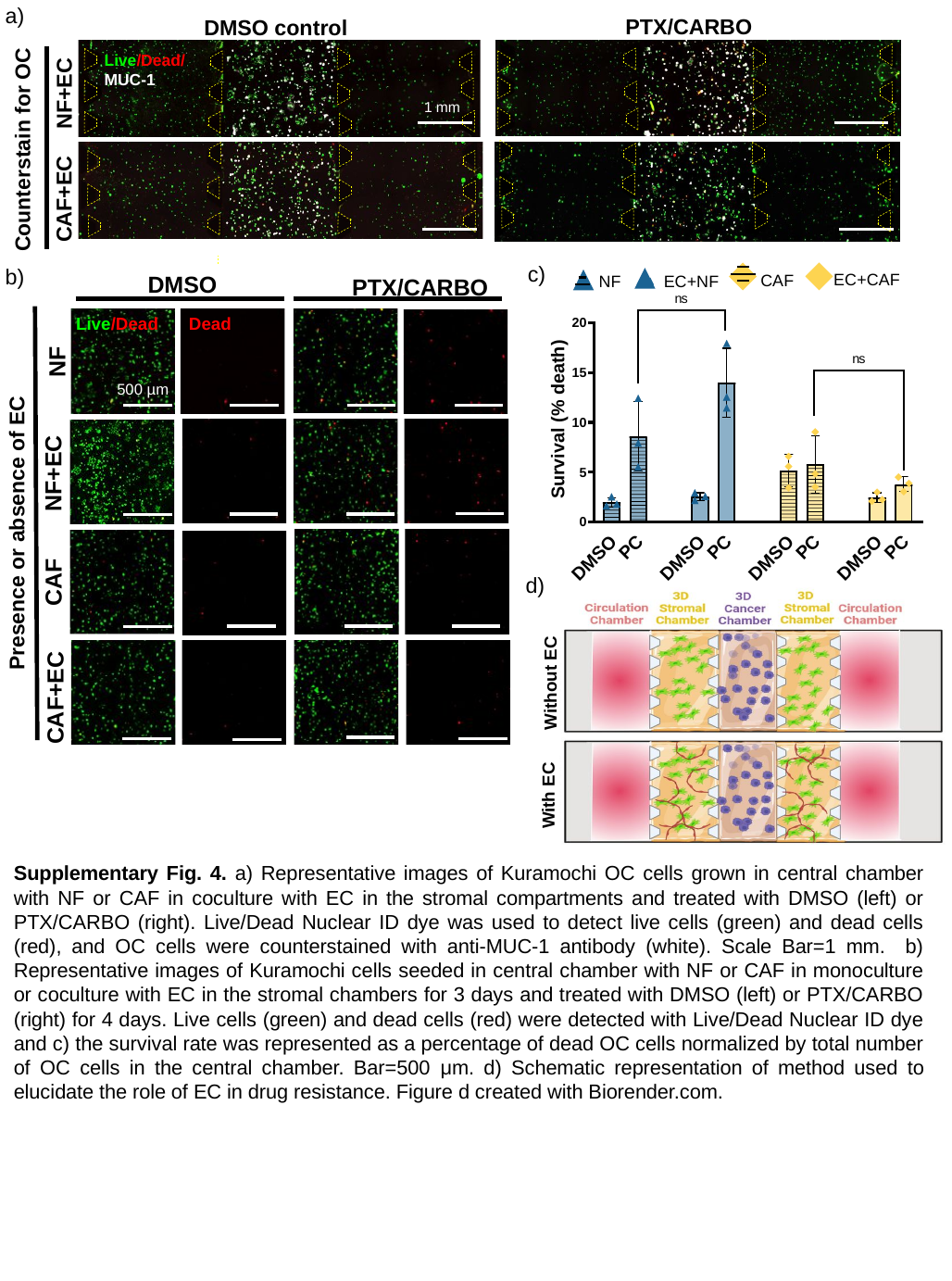

Healthy fibroblasts 090823
Counterstain for OC
a)
PTX/CARBO
DMSO control
NF+EC
CAF+EC
Live/Dead/
MUC-1
1 mm
c)
b)
NF
CAF
DMSO
PTX/CARBO
Live/Dead
Dead
Presence or absence of EC
500 µm
NF+EC
Without EC
d)
CAF+EC
With EC
Supplementary Fig. 4. a) Representative images of Kuramochi OC cells grown in central chamber with NF or CAF in coculture with EC in the stromal compartments and treated with DMSO (left) or PTX/CARBO (right). Live/Dead Nuclear ID dye was used to detect live cells (green) and dead cells (red), and OC cells were counterstained with anti-MUC-1 antibody (white). Scale Bar=1 mm. b) Representative images of Kuramochi cells seeded in central chamber with NF or CAF in monoculture or coculture with EC in the stromal chambers for 3 days and treated with DMSO (left) or PTX/CARBO (right) for 4 days. Live cells (green) and dead cells (red) were detected with Live/Dead Nuclear ID dye and c) the survival rate was represented as a percentage of dead OC cells normalized by total number of OC cells in the central chamber. Bar=500 μm. d) Schematic representation of method used to elucidate the role of EC in drug resistance. Figure d created with Biorender.com.

## Slide 5
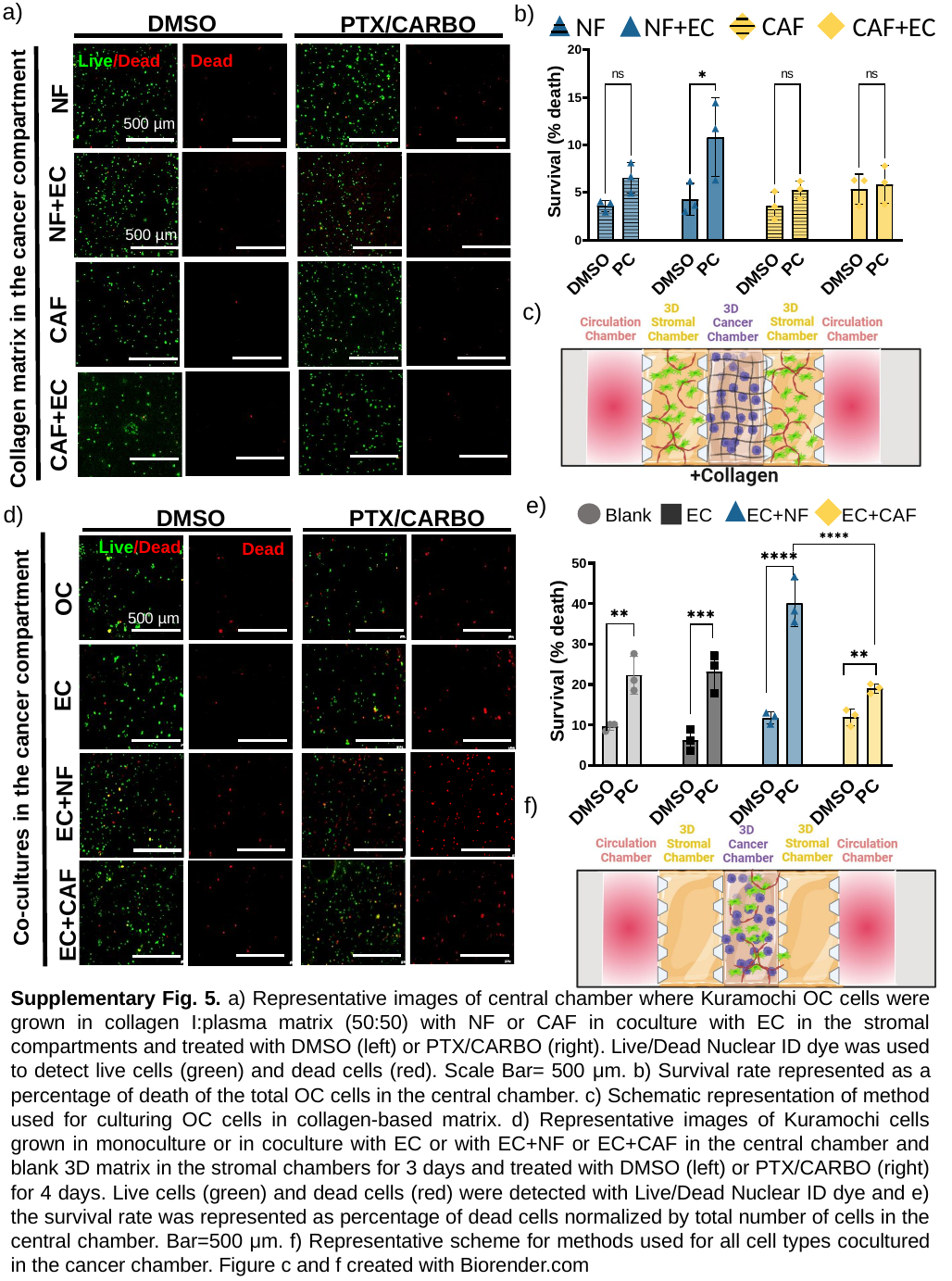

a)
b)
NF
CAF
CAF
NF+EC
CAF+EC
NF
DMSO
PTX/CARBO
Live/Dead
Dead
500 µm
NF+EC
CAF+EC
Collagen matrix in the cancer compartment
500 µm
c)
e)
d)
DMSO
PTX/CARBO
OC
EC
EC+NF
Live/Dead
Dead
500 µm
Co-cultures in the cancer compartment
f)
EC+CAF
Supplementary Fig. 5. a) Representative images of central chamber where Kuramochi OC cells were grown in collagen I:plasma matrix (50:50) with NF or CAF in coculture with EC in the stromal compartments and treated with DMSO (left) or PTX/CARBO (right). Live/Dead Nuclear ID dye was used to detect live cells (green) and dead cells (red). Scale Bar= 500 μm. b) Survival rate represented as a percentage of death of the total OC cells in the central chamber. c) Schematic representation of method used for culturing OC cells in collagen-based matrix. d) Representative images of Kuramochi cells grown in monoculture or in coculture with EC or with EC+NF or EC+CAF in the central chamber and blank 3D matrix in the stromal chambers for 3 days and treated with DMSO (left) or PTX/CARBO (right) for 4 days. Live cells (green) and dead cells (red) were detected with Live/Dead Nuclear ID dye and e) the survival rate was represented as percentage of dead cells normalized by total number of cells in the central chamber. Bar=500 μm. f) Representative scheme for methods used for all cell types cocultured in the cancer chamber. Figure c and f created with Biorender.com

## Slide 6
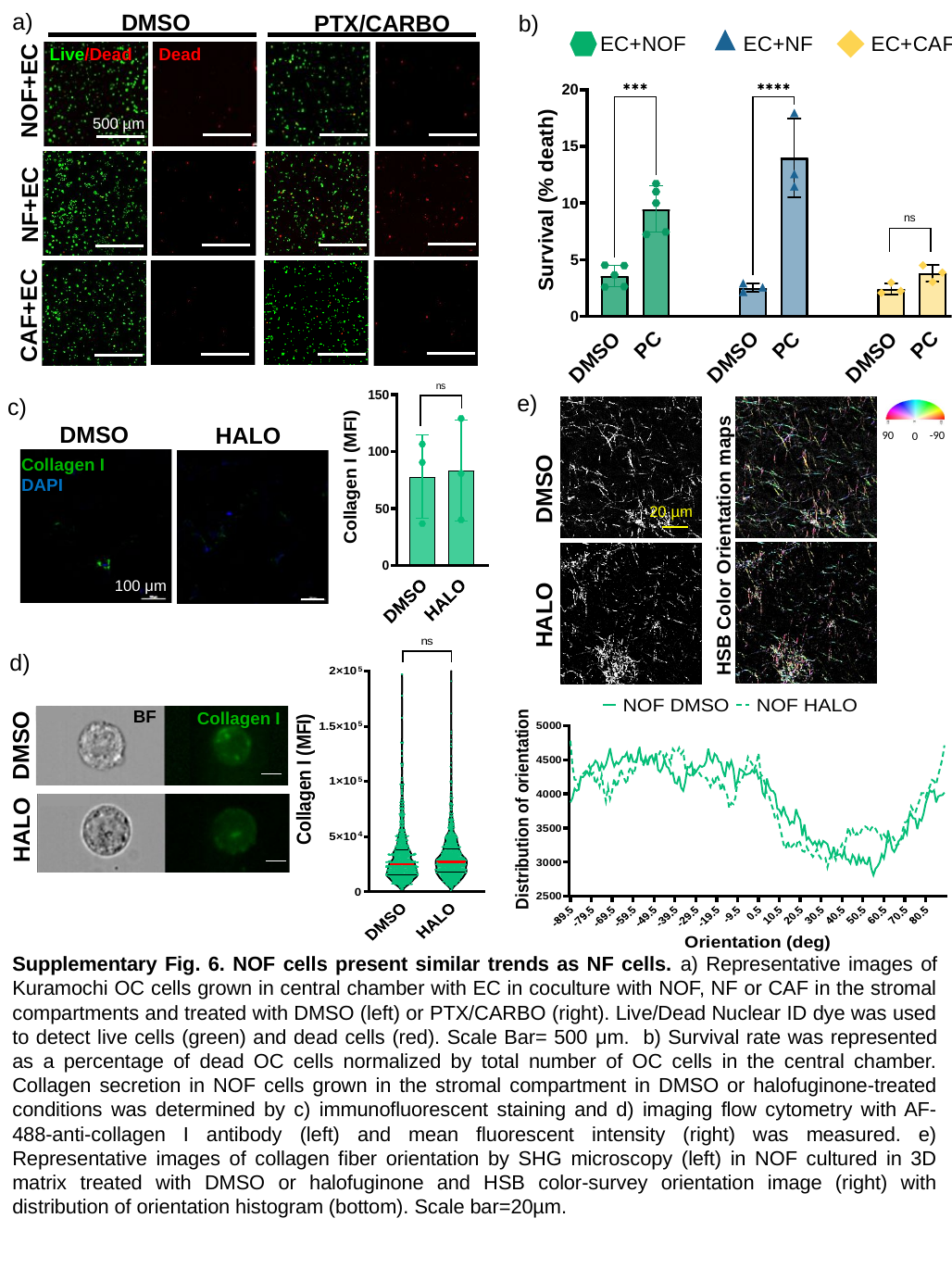

a)
DMSO
PTX/CARBO
b)
Live/Dead
Dead
NOF+EC
500 µm
NF+EC
CAF+EC
e)
c)
DMSO
HALO
-90
90
0
DMSO
Collagen I
DAPI
HSB Color Orientation maps
20 µm
HALO
100 μm
d)
DMSO
BF
Collagen I
HALO
Supplementary Fig. 6. NOF cells present similar trends as NF cells. a) Representative images of Kuramochi OC cells grown in central chamber with EC in coculture with NOF, NF or CAF in the stromal compartments and treated with DMSO (left) or PTX/CARBO (right). Live/Dead Nuclear ID dye was used to detect live cells (green) and dead cells (red). Scale Bar= 500 μm. b) Survival rate was represented as a percentage of dead OC cells normalized by total number of OC cells in the central chamber. Collagen secretion in NOF cells grown in the stromal compartment in DMSO or halofuginone-treated conditions was determined by c) immunofluorescent staining and d) imaging flow cytometry with AF-488-anti-collagen I antibody (left) and mean fluorescent intensity (right) was measured. e) Representative images of collagen fiber orientation by SHG microscopy (left) in NOF cultured in 3D matrix treated with DMSO or halofuginone and HSB color-survey orientation image (right) with distribution of orientation histogram (bottom). Scale bar=20µm.

## Slide 7
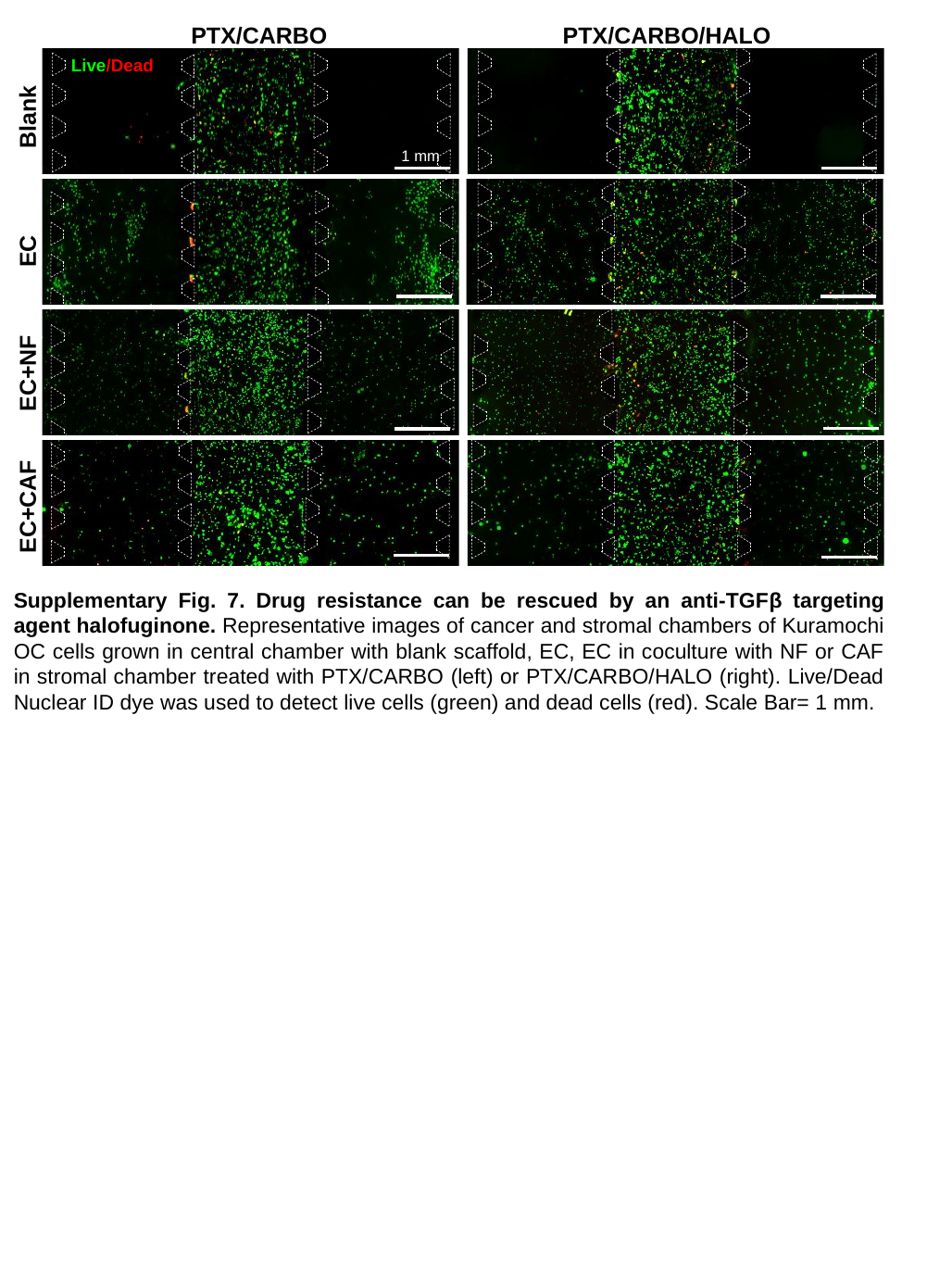

PTX/CARBO/HALO
PTX/CARBO
Live/Dead
Blank
1 mm
EC
EC+NF
EC+CAF
Supplementary Fig. 7. Drug resistance can be rescued by an anti-TGFβ targeting agent halofuginone. Representative images of cancer and stromal chambers of Kuramochi OC cells grown in central chamber with blank scaffold, EC, EC in coculture with NF or CAF in stromal chamber treated with PTX/CARBO (left) or PTX/CARBO/HALO (right). Live/Dead Nuclear ID dye was used to detect live cells (green) and dead cells (red). Scale Bar= 1 mm.
